# Bioclimatic and altitudinal variables influence the potential distribution of canine parvovirus type 2 worldwide

**DOI:** 10.1002/ece3.3994

**Published:** 2018-04-10

**Authors:** Feng Jiang

**Affiliations:** ^1^ College of Wildlife Resources Northeast Forestry University Harbin Heilongjiang Province China

**Keywords:** Canine parvovirus type 2, environmental variables, maximum entropy (MaxEnt), principal component analysis (PCA), risk

## Abstract

Canine parvovirus type 2 (CPV‐2) is extremely contagious and causes high rate of morbidity to many wild carnivores. It has three variants (CPV‐2a, CPV‐2b, and CPV‐2c) that are distributed worldwide with different frequencies and levels of genetic and antigenic variability. The disease poses a threat to the healthy survival and reproduction of wildlife. The research on the relationship between CPV‐2 epidemic and environmental variables is lacking. To fill this research gap, we used maximum entropy (MaxEnt) approach with principal component analysis (PCA) to evaluate the relation between CPV‐2 and environmental variables and to create a world risk map for this disease. According to the PCA results, 18 environmental variables were selected from 68 variables for subsequent analyses. MaxEnt showed that annual mean temperature, isothermality, altitude, November precipitation, maximum temperature of warmest month, and precipitation of warmest quarter were the six most important variables associated with CPV‐2 distribution, with a total of 77.7% percent contribution. The risk of this disease between 18°N and 47°N was high, especially in the east of China and the United States. These results support further prediction of risk factors for this virus to help secure the health and sustainable survival of wild carnivores.

## 1 INTRODUCTION

Canine parvovirus type 2 (CPV‐2) is a member of the genus *Parvovirus* of the family *Parvoviridae*. It was first identified in United States in the late summer of 1978 and subsequently found to have rapidly spread worldwide within 1–2 years (Carmichael, 2005) due to the lack of preexisting immunity in the canine population (Goddard & Leisewitz, 2010). This virus is extremely contagious that causes high morbidity, and mortality can reach 91% in untreated cases (National Center for Immunization and Respiratory Diseases, 2011).

CPV‐2 appeared initially as a new virus in the late 1970s (Appel, Scott, & Carmichael, 1979), and then, a series of new genetic and antigenic variants emerged which had increased in prevalence after a few years. CPV‐2a appeared in 1979, which replaced original CPV‐2 and regained the ability to infect cats and other carnivores (Truyen, Evermann, Vieler, & Parrish, 1996), and another variant called CPV‐2b was identified in 1984 in USA. Furthermore, in 2000, the third antigenic type called CPV‐2c was reported for the first time in Italy (Buonavoglia et al., 2001) and has been identified in many other European countries soon afterward (Decaro et al., 2006; Nakamura et al., 2004; Touihri et al., 2009), whereas this variant was more widespread in Asia (Nakamura et al., 2004) and America (Hong et al., 2007). CPV‐2a, CPV‐2b, and CPV‐2c are different from each other only by one amino acid residue (Shackelton, Parrish, Truyen, & Holmes, 2005). Epidemiological surveys indicate that the relative frequencies and genetic composition of them vary among countries (Decaro & Buonavoglia, 2012). Nevertheless, they have similar clinical signs (Wilson et al., 2014) ranging from mild to severe hemorrhagic enteritis, fever, vomiting, and often death in severe cases (Wilson et al., 2014).

Infections are normally acquired through contact with feces, vomit, saliva from infected dogs, and contaminated water or food. Upon entering the body, the virus replicates in the oropharynx in the first 2 days and transmits to other organs through bloodstream, and viremia appears after 3–5 days. The virus then reaches the lymphoid tissues, intestinal epithelium, and bone marrow, as well as the heart in neonatal pups, which affects mitotically active tissues. After an incubation period of 3–7 days, the disease can be characterized by either enteritis or myocarditis. In the intestine, the replication of the virus kills the embryonic epithelial cells of intestinal gland, leading to epithelial shedding, short villi, vomiting, hemorrhagic diarrhea, fever, dehydration, high degree of depression, shock, and even death, which are typical symptoms of enteric form, whereas myocarditis can be commonly seen in 4‐ to 6‐week‐old puppies, often without aura symptoms, or only mild diarrhea, moans, mucosal cyanosis, difficult breathing, fast and weak pulse, and often within a few hours suddenly death which probably due to acute respiratory depression. The characteristic of this disease is a very short clinical course with death that can often occur 2 or 3 days after onset of signs in nonprotected hosts (Carman & Povey, 1985). It can affect dogs at any age, but those puppies between 6 weeks and 6 months of age have the highest risk of developing severe disease (Houston, Ribble, & Head, 1996). While those dogs older than 1 year are still highly susceptible to CPV‐2 infection, they have a milder form and lower mortality of disease owing to discharging part of virus in feces (Wilson et al., 2014). CPV‐2 can also be transmitted directly to wild carnivores through close contact with domestic cats and dogs or via prey species of smaller carnivores (Miranda & Thompson, 2016).

Thus, CPV‐2 is not only one of the most significant enteric pathogens in domestic dogs and cats, but also has been detected in at least seven related families of wild carnivores (Decaro & Buonavoglia, 2012; Steinel, Parrish, Bloom, & Truyen, 2001), such as Grey wolf (*Canis lupus*; Allison et al., 2013), red fox (*Vulpes vulpes*; Filipov et al., 2016), Siberian tiger (*Panthera tigris altaica*; Steinel, Munson, van Vuuren, & Truyen, 2000), Masked palm civet (*Paguma larvata*; Chen et al., 2011), Red panda (*Ailurus fulgens*), and Giant panda (*Ailuropoda melanoleuca*; Mainka, Qiu, He, & Appel, 1994). While the disease poses a potential threat to wild carnivore survival and reproduction in many countries around the world, it remains unclear what environmental conditions influence the incidence of the disease in the wild. So, epidemiological surveillance and risk predictions require additional insight about environmental factors that determine the geographic distribution of the disease.

Studies have shown that environmental factors such as geography, climate, and weather have a significant influence on the geographic distribution of animal viruses. For example, adenovirus (Fagbo et al., 2016), rotavirus (Das et al., 2017), respiratory syncytial virus (Nenna et al., 2017), hantavirus (Prist, Uriarte, Fernandes, & Metzger, 2017; Tian et al., 2017), and avian influenza virus (Tian et al., 2015) are closely related to temperature, precipitation, and humidity, which may vary locally and seasonally (Lujan, Greenberg, Hung, Dimenna, & Hofkin, 2014). Indeed, CPV‐2 shows local and seasonal characteristics (Schoeman, Goddard, & Leisewitz, 2013; Zhao et al., 2016). But how environmental conditions and CPV‐2 incidence are related reminds uncertain. Here, I report on an analysis that links environmental characteristics with CPV‐2 incidence across the globe. I applied maximum entropy (MaxEnt) analysis, a kind of ecological niche modeling (Phillips et al., 2009), to ascertain the association between geospatial variation in environmental factors and geospatial patterns of CPV‐2 incidence. The study aims to provide a basis for developing early warning predictions of when and where canine parvovirus is like to emerge.

## 2 MATERIALS AND METHODS

I used MaxEnt to model the association between CPV‐2 distributing and environmental variables. MaxEnt is a machine learning method that estimates species distributions by finding the probability distribution using the maximum entropy principle with constraints on the expected values of the environmental predictors (Phillips, Anderson, & Schapire, 2006). It requires only presence records of the species and remains effective despite small sample size (Padalia, Srivastava, & Kushwaha, 2015). Moreover, this method combines species occurrence data and spatial environmental variables to produce an index of relative suitability that varies from 0 (unsuitable or most dissimilar to presence locations) to 1 (most suitable or most similar to presence locations; Kumar et al., 2015). I reduce the likelihood of aliasing between environment variables and eliminate highly correlated variables using principle component analysis (PCA; Freeman, Kleypas, & Miller, 2013).

### 2.1 Canine parvovirus 2 data collection

Geographical coordinates of known CPV‐2 records were obtained using *canine parvovirus type 2* as search terms and downloading the global CPV‐2 gene sequence information in GenBank (https://www.ncbi.nlm.nih.gov/genbank/). Further literature searches were made in PubMed according to the title or PubMed Unique Identifier found in the gene sequence supplemental information.

Google Earth software was used to obtain the geographical coordinates of the given city, town, or village in which CPV‐2 was reported, whenever exact geographical coordinates were not provided (Miller et al., 2012). Studies for which such information could not be obtained excluded. This produced 549 geographical coordinates from GenBank, and 285 from PubMed, for a total of 834 geospatial locations.

I used a regular grid with 1 km × 1 km cells analyzed by ArcGis10.2 software, in order to make a maximum of one distribution point in each grid cell, thereby eliminating duplicate or very close record points. This meant that 228 CPV‐2 geographical coordinates were excluded, leaving 606 as inputs for MaxEnt. All data were entered into a single spreadsheet file and saved as “.csv” format.

### 2.2 Environmental variables data

Environmental data were obtained from WorldClim‐Global Climate Data (Available from: http://www.worldclim.org/). WorldClim environmental variables were obtained from weather stations averaged over a 50‐year period (from 1950 to 2000) at the 30 arc‐seconds (~1 km) spatial resolution. I converted those data to “.asc” format required by MaxEnt software (Syfert, Smith, & Coomes, 2013). Of the 68 variables considered, 48 were climate variables that describe monthly total precipitation and average, minimum, and maximum monthly temperature; the remaining 20 were 19 bioclimatic variables and one altitude.

### 2.3 Statistical analysis

Multivariate statistical analyses require using explanatory variables that are not closely correlated (Syfert et al., 2013; Zuur, Ieno, & Elphick, 2010). I therefore removed the potential for collinearity among correlated variables using PCA such that the correlation coefficients among the variables used in the analyses were <0.80 (Freeman et al., 2013; Kumar et al., 2015).

I used ArcGis10.2 software to convert CPV‐2 point data to shape raster and extract the attribute values of the 68 environment variables of 606 records points by “extract the analysis tool” from spatial analyst, then exported the attribute value data to the “txt” text file and convert spreadsheet file, and finally entered the data into the software to calculate and analyze. All calculations were made in SPSS 22.

### 2.4 Ecological Niche Modeling

MaxEnt 3.3.3k version (http://www.cs.princeton.edu/~schapire/maxent/) was used on computer running Java version 1.4 or later, and the Java runtime environment was obtained from http://www.oracle.com/technetwork/java/javase/downloads/index-jsp-138363.html.

Occurrence points were divided randomly into training data and test data. Of these points, 75% were utilized as training data for model prediction and 25% were used as test data for model testing and independent validation purposes. I used a Jackknife procedure to assess the contribution of each variable to model prediction. This procedure was replicated 10 times (Johnson et al., 2016; Miller et al., 2012). The best fit model was judged using the area under the receiver operating characteristic (ROC) curve (AUC; Jiang et al., 2016).

ROC curves relate true positive rate against false‐positive error rate on an xy‐coordinate system. The AUC (the area under ROC curve) value ranges between 0 and 1. Higher values indicated better model performance (Fourcade, Engler, Rödder, & Secondi, 2014). Hence, a model is judged not to perform better than random if the AUC is below 0.5, and generally model performance is considered high when AUC values exceed 0.9.

## 3 RESULTS

### 3.1 Determination of environmental variables

**Figure 1:**
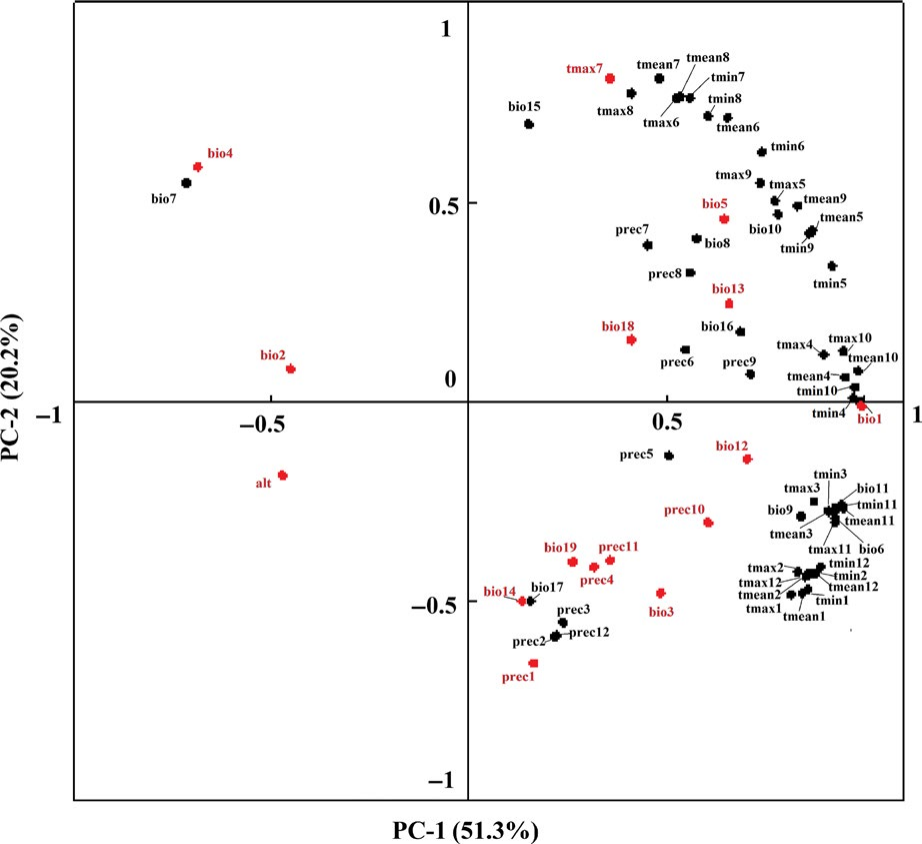
Plot of PC‐1 and PC‐2 scores of environmental variables of CPV‐2. The 68 environmental variables initially considered as projected into principle component space in this study. Each vector group is detailed in Table S2

Principle component analysis revealed that much of the variation in environmental variables could be explained by the first and second principal component (PC1 and PC2) which explained about 71.5% of the total variance of the environmental variables data (Figure 1). The PC1 summarized more than 51.3% of the information, which was temperature variable. It can therefore be interpreted as a temperature factor. The PC2, summarizing 20.2% of the information, was a combination of temperature and precipitation variables. Overall, PCA identified 18 groups of highly correlated variables with each group having more than one variable. I thus selected only one variable from each group in final calculations, reducing the number of explanatory environmental variables considered from 68 to 18 (Table 1).

**Table 1:** Statistic analysis of environmental variables with small correlation coefficients

Code	Variables	IEV	Max	Min	Mean	*SD*	OR
Alt (m)	Altitude	–	4,202	1	459.36	781.42	0~300
Bio1 (°)	Annual mean temperature	*T*	28.6	−3.9	15.70	6.71	8.5~16
Bio2 (°)	Mean diurnal range	*T*	19.3	4.4	10.57	2.54	10.4~13.4
Bio3	Isothermality (bio2/bio7)(×100)	*T*	85	20	36.72	12.12	20~36
Bio4	Temperature seasonality (standard deviation × 100)	*T*	15,875	239	7,059.92	3,290.21	4,000~5,000
Bio5 (°)	Max temperature of warmest month	*T*	41.8	13.8	30.70	4.08	27.5~33.5
Bio8 (°)	Mean temperature of wettest quarter	*T* *+* *P*	30.6	1.9	22.15	6.05	25.5~27.5
Bio12 (mm)	Annual precipitation	*P*	3,216	54	1016	537.17	1,000~1,100
Bio13 (mm)	Precipitation of wettest month	*P*	653	13	198.88	112.95	100‐200
Bio14 (mm)	Precipitation of driest month	*P*	195	0	25.68	29.67	0~15
Bio15	Precipitation seasonality (coefficient of variation)	*P*	148	7	70.23	34.40	90~100
Bio18 (mm)	Precipitation of warmest quarter	*T + P*	1,639	5	423.96	288.65	200~500
Bio19 (mm)	Precipitation of coldest quarter	*T + P*	796	4	118.79	130.71	0~50
Prec1 (mm)	January precipitation	*P*	264	1	41.86	49.05	0~15
Prec4 (mm)	April precipitation	*P*	251	2	67.05	46.0	15~45
Prec10 (mm)	October precipitation	*P*	385	2	71.23	60.47	25~50
Prec11 (mm)	November precipitation	*P*	332	1	52.93	56.40	20~50
Tmax7 (°)	July maximum temperature	*T*	36.9	11.9	28.61	5.34	31~32.5

IEV is initial environmental variables, Min. is minimum, Max. is maximum, *SD* is standard deviation, *C·V* is coefficient of variation, and OR is optimum range. Bioclimatic variables computed from temperatures (*T*), from precipitation sums (*P*), or from both (*T* + *P*).

The 18 environmental variables essentially represent three categories, namely temperature‐related, precipitation‐related, and terrain‐related environmental variables (Table 1). The correlation coefficient between them was less than 0.8 (Freeman et al., 2013; Kumar et al., 2015; Table S1).

### 3.2 Model evaluation and environmental variable importance

**Figure 2:**
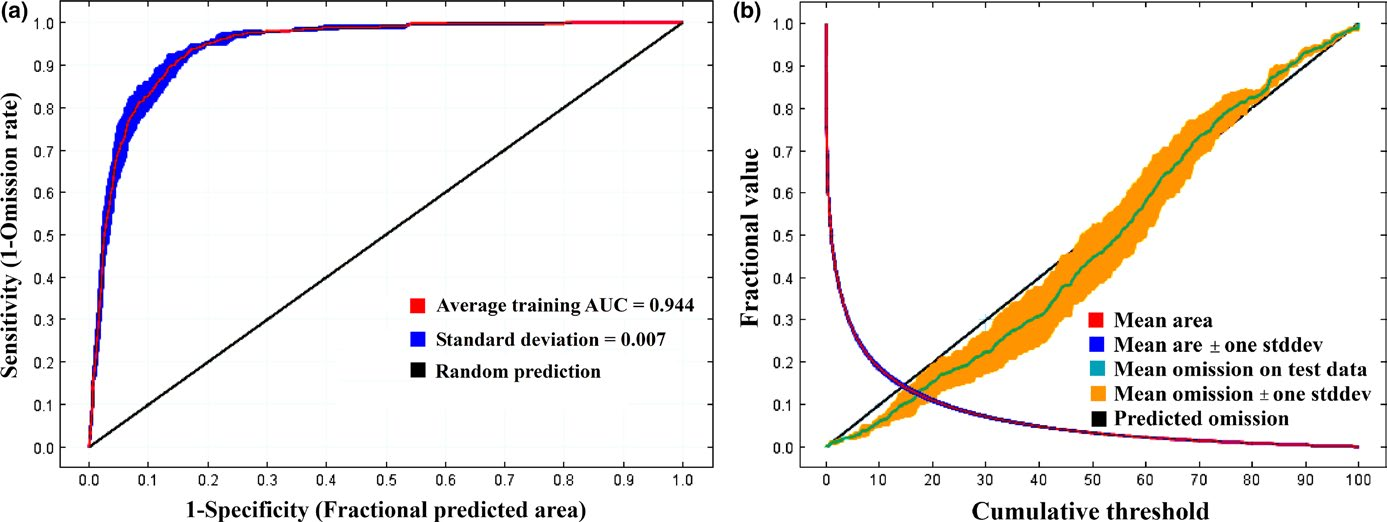
Statistical charts of MaxEnt analysis, (a) ROC and AUC of prediction, and (b) the omission and predicted area, where the values indicate the training gain with only variables

The 18 candidate environmental variables (Table 1) were used as input for the MaxEnt model. The mean AUC_test_ for the 10 replicate models of CPV‐2 was 0.949, and the standard deviation was 0.007 with low omission rates and *p*‐values (Figure 2), which indicates that MaxEnt model had a high accuracy.

The relative contribution of environmental variables in predictive species distribution models is evaluated utilizing the jackknife test in MaxEnt, which indicates that annual mean temperature (Bio 1), isothermality (Bio3), altitude (Alt), November precipitation (Prec11), maximum temperature of warmest month (Bio5), and precipitation of warmest quarter (Bio18) were the most important environmental variables associated with CPV‐2 distribution, with a total of 77.7% contribution. Among them, annual mean temperature was the top most important predictor which contributed 21.8% and it had the most information that was not present in other variables. Moreover, there were 28 environmental variables with high correlation with annual mean temperature, including min temperature of coldest month, mean temperature of driest quarter, mean temperature of coldest quarter, temperature (maximum temperature, minimum temperature, and average temperature) from January to May and October to December, and September average temperature (Table S2). As shown in the table (Table S2), it showed that CPV‐2 presence was higher at lower levels of monthly and seasonal temperature. It is also possible to conclude that CPV‐2 is more closely related to temperature.

However, mean diurnal range (Bio2), temperature seasonality (Bio4), January precipitation (Prec1), precipitation of coldest quarter (Bio19), July maximum temperature (Tmax7), and precipitation of driest month (Bio14) were relatively the six least important environmental variables, with just 4.9% contribution in total.

**Figure 3:**
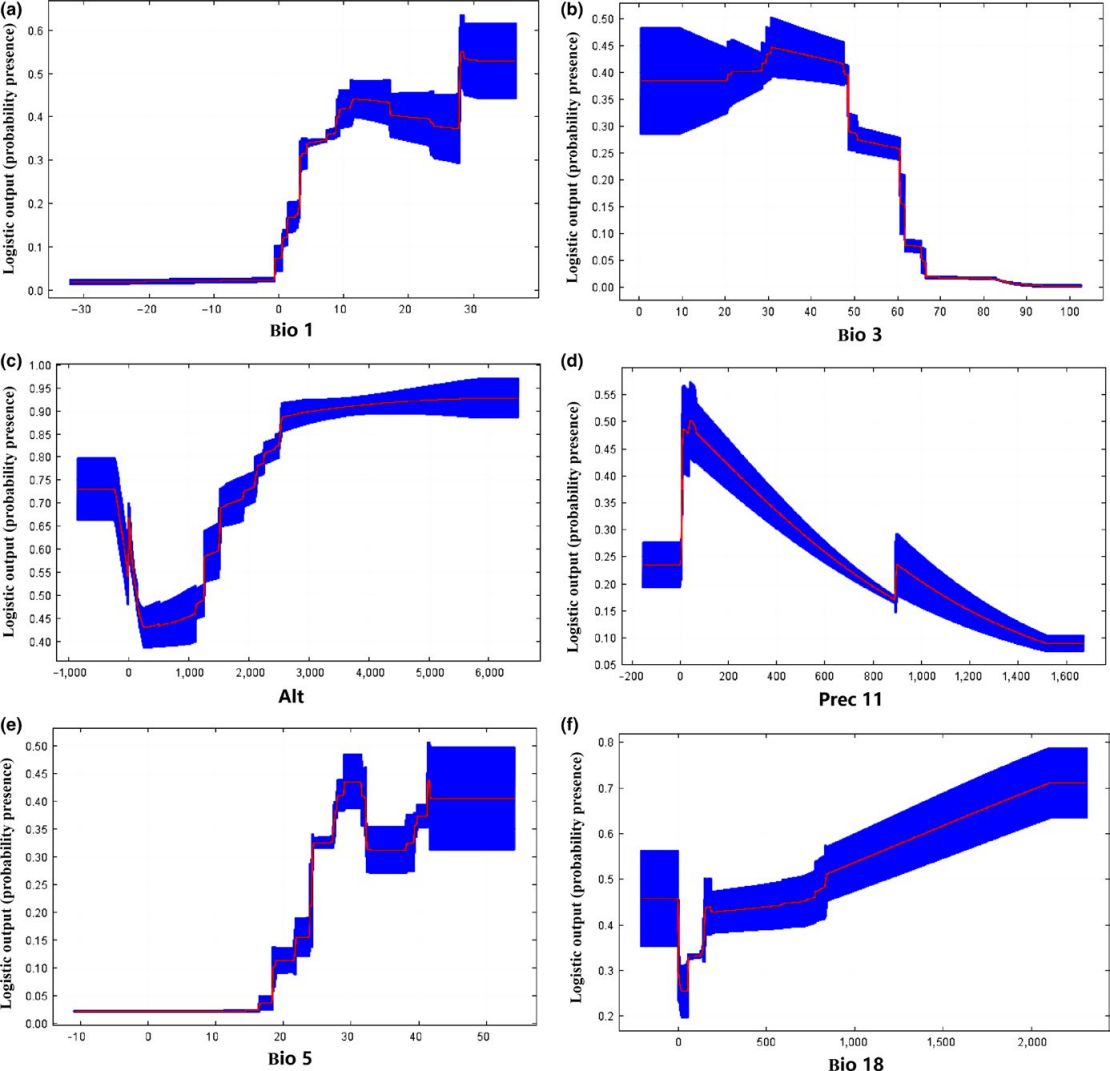
Relationships between top environmental predictors and the probability of presence of CPV‐2. (a) Annual mean temperature (°C). (b) Isothermality (BIO2/BIO7 × 100). (c) Altitude (m). (d) November precipitation (mm). (e) Maximum temperature of warmest month (°C). (f) Precipitation of warmest quarter (mm)

**Figure 4:**
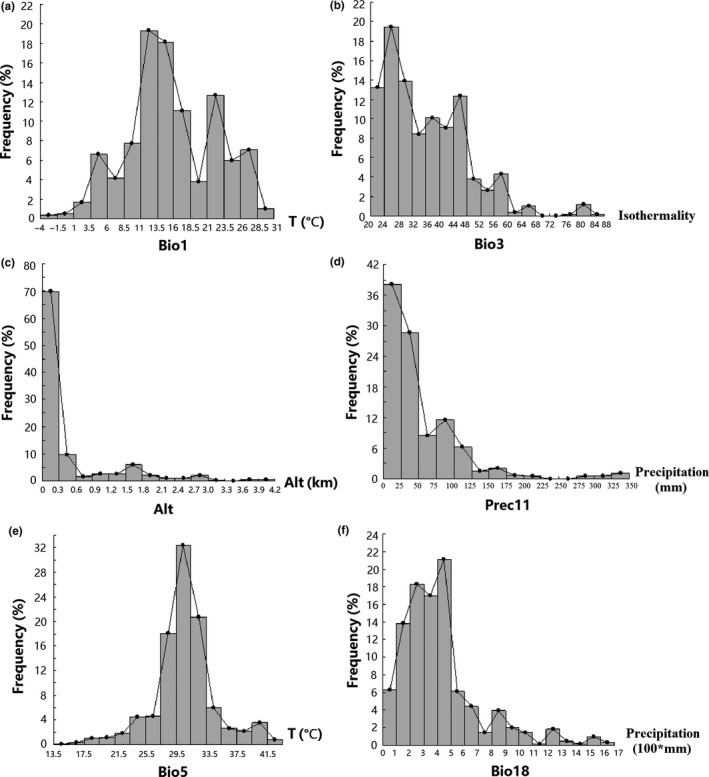
Frequency distribution of six environment variables with contribution rates with a relatively high contribution rate. (a) Annual mean temperature (°C). (b) Isothermality (BIO2/BIO7 × 100). (c) Altitude (m). (d) November precipitation (mm). (e) Maximum temperature of warmest month (°C). (f) Precipitation of warmest quarter (mm)

Response curves were created automatically by MaxEnt which explained the environmental preferences of CPV‐2. The six most important variables were analyzed (Figure 3a–f) combined with frequency distributions (Figure 4a–f). It shows that altitude was negatively correlated with CPV‐2 distribution and below 300 m was favorable for CPV‐2 (Figures 3c, 4c, Table 1), while the other five environmental variables were positively correlated. Among the three variables related to temperature, annual mean temperature ranged from −3.9 to 28.6°C and the optimum range was 8.5–16°C (Figure 3a, Table 1). And CPV‐2 currently occurs in areas with isothermality between 20 and 85, and a maximum temperature of warmest month between 13.8 and 41.8°C. However, the optimum ranges of them were 20–36 and 27.5–33.5°C, respectively (Figures 3b,e and 4b,e, Table 1). Moreover, November precipitation and precipitation of warmest quarter were two variables related to rainfall, and the optimum range of them was 20‐50 mm and 200‐500 mm, respectively (Figures 3d,f, and 4d,f, Table 1).

### 3.3 Spatial distribution of CPV‐2 Risk

**Figure 5:**
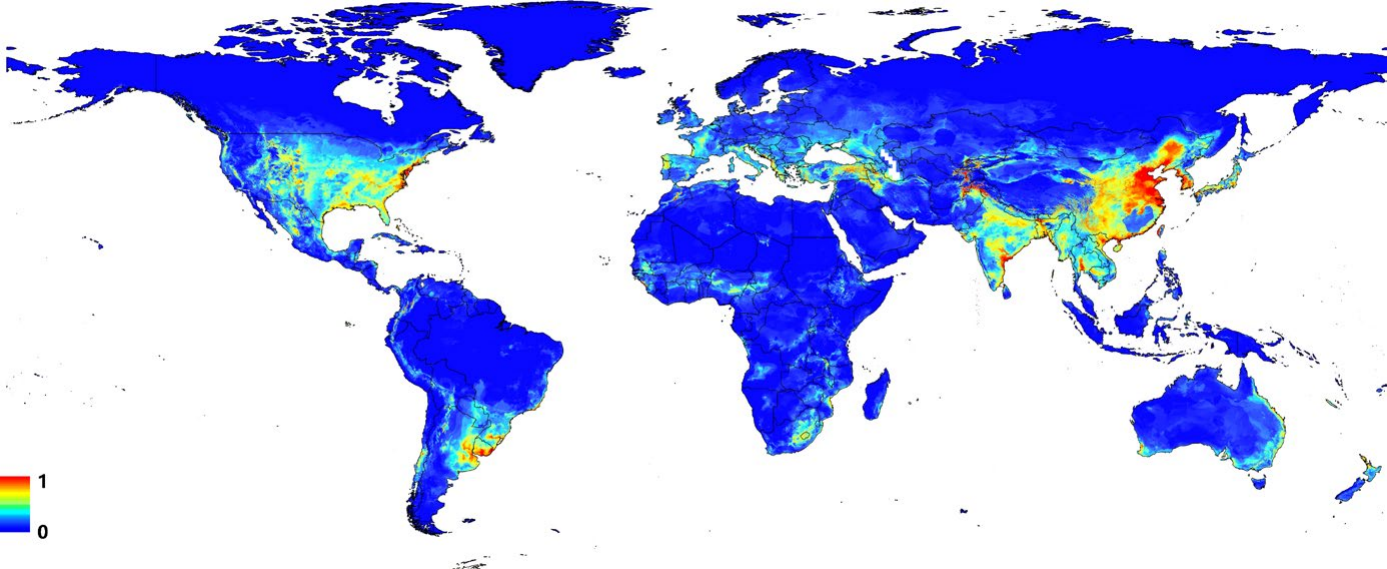
Predicted potential geographic distributions for CPV‐2 in the world. Color scale indicates the probability that conditions are the risk level for CPV‐2: red = high‐risk probability, green = average‐risk probability, blue = low‐risk probability

The MaxEnt model reveals that high CPV‐2 incidence risk occurs in the eastern part of Asia (including the central and eastern coastal areas of China, Japan, Korea, Korea), the southern (including most of India, Bangladesh, Myanmar, southern Thailand, northeastern Vietnam, central Cambodia; including central and eastern parts of Central America and eastern), Europe (central and northern Portugal, central France and Italy), southern North America (including central and eastern United States, northern Mexico), and South America (including eastern Argentina, Uruguay, southern Brazil And coastal, southern Chile; Figure 5).

## 4 DISCUSSION

It has now been about 30 years since CPV‐2 emerged; however, the disease caused by CPV‐2 was not recognized until serious or fatal illness affected large numbers of dogs and other canids.

What remains uncertain, however, is what environmental factors determine its global distribution. My analysis revealed that temperature, precipitation, and altitude have an effect on the distribution of CPV‐2, more specifically annual mean temperature, isothermality, altitude, November precipitation, maximum temperature of warmest month, and precipitation of warmest quarter were the most important environmental variables for CPV‐2.

With regard to terrain, CPV‐2 cases are expected to occur mainly in low altitude areas of <300 m (Table 2, Figures 3c, and 4c). This is based on the fact that 69.8% of collected data used in the analysis were collected from below 300 meters, with 51.8% coming from below 100 m.

**Table 2:** Relative importance of environmental variables in MaxEnt model

Variable	Percent contribution	Variable	Percent contribution
Bio 1	**21.8**	Bio 15	1.8
Bio 3	**15.6**	Bio 8	1.7
Alt	**15.4**	Prec 10	1.5
Prec 11	**11.3**	Bio 2	1.1
Bio 5	**7.2**	Bio 4	1.0
Bio 18	**6.4**	Prec 1	1.0
Bio 13	5.6	Bio 19	0.8
Bio 12	4.2	Tmax 7	0.5
Prec 4	2.6	Bio 14	0.5

Variables with high percent contribution are indicated in bold.

Moreover, the incidence of CPV‐2 was significantly higher in the season with high temperature difference (Fu, Pei, Wang, & Yin, 2012). Approximately 48.5% of 606 cases had an annual mean temperature of 8.5–16°C, and 55% had isothermality of 20–36. From the perspective of environmental variables of precipitation, it indicated that in the case of occurrence, about 66.8% of the November precipitation was in the range of 20–50 mm and about 56.4% of the precipitation of warmest quarter was in the range of 200–500 mm.

Statistics also showed that CPV‐2 cases were prone to occur in low altitudes, but for global cases, it can still occur at high altitudes or even high altitudes. CPV‐2 infected a lot of host types, and their living areas are also different. For example, Grey wolf can live in plains, deserts, hills, mountains, and even high mountains (such as Himalayas) areas. Siberian tigers live mainly in mountainous areas. Puma (*Puma concolor*) can live in forests, jungles, hills, and grasslands. Giant pandas are also mainly in the mountains and valleys in the upper reaches of the Yangtze River in China, living in the 2,600~3,500 m above sea level in the dense bamboo forest. At the same time, the contribution rate of altitude to CPV‐2 was relatively large (Table 2). It can be seen that the host of CPV‐2 has a wide distribution and large terrain difference, and thus, the altitude varied greatly (Table 1).

Globally, the high risk of CPV‐2 prediction is mainly in the eastern and northern parts of Asia in the range of 20°N to 45°N, which is consistent with the actual occurrence of the case. High risk is mainly in the central and eastern coastal areas in China.

This study used the maximum entropy to forecast the potential risk of CPV‐2 in a global scale, which shows how an ecological approach can help to improve our understanding of the geographic distribution of CPV‐2 incidence, as well as the association between this global distribution and geospatial variation in environmental factors. Risk prediction maps can provide guidance on the protection of wild animals in high‐risk areas. For example, Giant Panda Sanctuaries in Sichuan, Shaanxi, and Gansu Provinces, China, are at a high risk of CPV‐2, where there are many wild animals susceptible to the disease such as *Ailurus fulgens*,* Nyctereutes procyonoides,* and *Procyon lotor*, so that they are most likely to be infected with each other through the close contact or fomites. And the red panda in Yunnan Provinces, China, and the Grey wolf in northern USA may also face the threat of CPV‐2. Therefore, it is essential to pay close attention to the high‐risk area of CPV‐2, especially wildlife reserves in various countries, and it is necessary to monitor climate data and contact with domestic animals in these regions simultaneously.

## CONFLICT OF INTEREST

None declared.

## AUTHOR CONTRIBUTION

FJ conceived and designed the experiments, collated data, performed modeling work, analyzed the output data, and wrote the first draft of the manuscript.
