# Supplementary material for: Bioclimatic and altitudinal variables influence the potential distribution of canine parvovirus type 2 worldwide
Source: Ecol Evol. 2018 Apr 10;8(9):4534–43. doi: 10.1002/ece3.3994 (PMC5938446; doi:10.1002/ece3.3994)
Supplement: Supplementary file 1 [file ECE3-8-4534-s001.doc]

**TABLE S1** Correlation matrix of variables selected for species distribution model analysis. Variables are listed along the top and left side, with ρ-correlations for each variable pair given in the table.

|  | Alt | Bio1 | Bio2 | Bio3 | Bio4 | Bio5 | Bio8 | Bio12 | Bio13 | Bio14 | Bio15 | Bio18 | Bio19 | Prec1 | Prec4 | Prec_10 | Prec11 | Tmax7 |
| --- | --- | --- | --- | --- | --- | --- | --- | --- | --- | --- | --- | --- | --- | --- | --- | --- | --- | --- |
| Alt | 1.00 |  |  |  |  |  |  |  |  |  |  |  |  |  |  |  |  |  |
| Bio1 | -0.45 | 1.00 |  |  |  |  |  |  |  |  |  |  |  |  |  |  |  |  |
| Bio2 | 0.55 | -0.38 | 1.00 |  |  |  |  |  |  |  |  |  |  |  |  |  |  |  |
| Bio3 | 0.27 | 0.52 | 0.19 | 1.00 |  |  |  |  |  |  |  |  |  |  |  |  |  |  |
| Bio4 | -0.01 | -0.69 | 0.33 | -0.78 | 1.00 |  |  |  |  |  |  |  |  |  |  |  |  |  |
| Bio5 | -0.47 | 0.69 | 0.07 | 0.12 | -0.03 | 1.00 |  |  |  |  |  |  |  |  |  |  |  |  |
| Bio8 | -0.49 | 0.58 | -0.32 | 0.05 | -0.12 | 0.54 | 1.00 |  |  |  |  |  |  |  |  |  |  |  |
| Bio12 | -0.36 | 0.61 | -0.57 | 0.26 | -0.52 | 0.21 | 0.31 | 1.00 |  |  |  |  |  |  |  |  |  |  |
| Bio13 | -0.33 | 0.58 | -0.46 | 0.20 | -0.36 | 0.30 | 0.48 | 0.80 | 1.00 |  |  |  |  |  |  |  |  |  |
| Bio14 | -0.25 | 0.08 | -0.28 | -0.03 | -0.16 | -0.08 | -0.13 | 0.51 | -0.02 | 1.00 |  |  |  |  |  |  |  |  |
| Bio15 | 0.01 | 0.17 | 0.12 | -0.02 | 0.18 | 0.33 | 0.45 | -0.10 | 0.43 | -0.72 | 1.00 |  |  |  |  |  |  |  |
| Bio18 | -0.27 | 0.31 | -0.49 | 0.01 | -0.19 | 0.02 | 0.45 | 0.74 | 0.76 | 0.17 | 0.21 | 1.00 |  |  |  |  |  |  |
| Bio19 | -0.11 | 0.23 | -0.16 | 0.16 | -0.28 | 0.10 | -0.31 | 0.44 | 0.04 | 0.60 | -0.45 | -0.04 | 1.00 |  |  |  |  |  |
| Prec1 | -0.10 | 0.14 | -0.14 | 0.25 | -0.31 | -0.06 | -0.32 | 0.37 | -0.08 | 0.68 | -0.57 | 0.00 | 0.64 | 1.00 |  |  |  |  |
| Prec4 | -0.18 | 0.24 | -0.45 | 0.12 | -0.31 | -0.03 | -0.06 | 0.66 | 0.21 | 0.67 | -0.53 | 0.37 | 0.61 | 0.66 | 1.00 |  |  |  |
| Prec10 | -0.24 | 0.58 | -0.35 | 0.42 | -0.58 | 0.24 | 0.23 | 0.58 | 0.33 | 0.44 | -0.24 | 0.12 | 0.34 | 0.30 | 0.31 | 1.00 |  |  |
| Prec11 | -0.22 | 0.34 | -0.24 | 0.24 | -0.36 | 0.16 | -0.07 | 0.38 | 0.09 | 0.50 | -0.39 | -0.05 | 0.50 | 0.57 | 0.35 | 0.72 | 1.00 |  |
| Tmax7 | -0.25 | 0.35 | 0.01 | -0.20 | 0.28 | 0.71 | 0.37 | 0.16 | 0.32 | -0.20 | 0.45 | 0.16 | -0.01 | -0.25 | -0.04 | 0.04 | -0.01 | 1.00 |

**TABLE S2** Groups of highly correlated variables from principal components analysis. Variable selected for subsequent analyses is indicated in bold

|  | Highly correlated variables | Total |
| --- | --- | --- |
| **Alt** | - | 0 |
| **Bio1** | Bio6, Bio9, Bio11, Tmax1~5, Tmax10~12, Tmean1~5, Tmean9~12, Tmin1~5, Tmin10~12 | 28 |
| **Bio2** | - | 0 |
| **Bio3** | - | 0 |
| **Bio4** | Bio6~7, Bio11, Tmax1~2, Tmax11~12, Tmean1~3, Tmean11~12, Tmin1~3, Tmin11~12 | 17 |
| **Bio5** | Bio10, Tmax5~6, Tmax9, Tmean5 | 5 |
| **Bio8** | - | 0 |
| **Bio12** | Bio16,Prec5, Prec6,Prec9 | 4 |
| **Bio13** | Bio16, Prec6~8 | 4 |
| **Bio14** | Bio17 | 1 |
| **Bio15** | - | 0 |
| **Bio18** | Bio16, Prec8 | 2 |
| **Bio19** | - | 0 |
| **Prec1** | Prec2, Prec3, Prec12 | 3 |
| **Prec4** | Prec3 | 1 |
| **Prec10** | - | 0 |
| **Prec11** | Prec12 | 1 |
| **Tmax7** | Tmax6, Tmax8, Tmax9, Tmean6~8, Tmin6~8 | 9 |
